# Supplementary material for: MetaRibo-Seq measures translation in microbiomes
Source: Nat Commun. 2020 Jun 29;11:3268. doi: 10.1038/s41467-020-17081-z (PMC7324362; doi:10.1038/s41467-020-17081-z)
Supplement: Supplementary file 10 — Supplementary Data 7 [file 41467_2020_17081_MOESM10_ESM.zip › File2/Confidence_High_Taxonomy/377968_out.krona.html]

Javascript must be enabled to view this page.

members
magnitude
magnitudeUnassigned
count
unassigned
taxon
rank

377968\_out

31

2
31
superkingdom

phylum
31
976

class
200643
31

order
31
171549

171552
31
family

genus
838
31

24
165179

SRS013098\_contig\_number\_50627SRS014415\_contig\_number\_contig-100\_459.27312SRS015782\_contig\_number\_47141SRS015794\_contig\_number\_11057SRS018888\_contig\_number\_9683SRS019276\_contig\_number\_4922SRS019285\_contig\_number\_contig-100\_198.22088SRS043841\_contig\_number\_2836SRS043841\_contig\_number\_4533SRS047044\_contig\_number\_1912SRS047433\_contig\_number\_5461SRS053398\_contig\_number\_23235SRS065397\_contig\_number\_contig-100\_1449.92496SRS077641\_contig\_number\_3162SRS077991\_contig\_number\_153SRS078419\_contig\_number\_3994SRS104036\_contig\_number\_6934SRS104327\_contig\_number\_2666SRS143181\_contig\_number\_13923SRS144506\_contig\_number\_4278SRS144603\_contig\_number\_contig-100\_2006.96130SRS146764\_contig\_number\_12339SRS147614\_contig\_number\_3390SRS148253\_contig\_number\_14123
species

species

SRS012969\_contig\_number\_1527SRS012989\_contig\_number\_contig-100\_2158.56858.56858SRS013098\_contig\_number\_50626SRS013638\_contig\_number\_contig-100\_388.123927SRS023526\_contig\_number\_21899SRS064757\_contig\_number\_3527SRS077849\_contig\_number\_27783
7
59823
